# Tracheal Adenoid Cystic Carcinoma Successfully Treated With the Combined Core Out Technique, Cryoextraction, and Argon Plasma Coagulation: A Case Report

**DOI:** 10.7759/cureus.64150

**Published:** 2024-07-09

**Authors:** Parinya Ruenwilai, Kulachat Ekpumimas

**Affiliations:** 1 Division of Pulmonary and Critical Care, Department of Internal Medicine, Phramongkutklao Hospital, Bangkok, THA; 2 Division of Respiratory Medicine, Central Chest Institute of Thailand, Nonthaburi, THA

**Keywords:** malignant airway obstruction, tracheal adenoid cystic carcinoma, tracheal tumor, pulmonary adenoid cystic carcinoma, adenoid cystic carcinoma

## Abstract

We report the case of a 67-year-old male who presented with mild dyspnea two years ago, with increasing intensity, cough, and stridor on exertion. He underwent outpatient evaluation and received treatment for recurrent episodes of bronchitis and acute exacerbations of chronic obstructive pulmonary disease. His current medication included tiotropium 18 µg per day and salmeterol/fluticasone 50/500 µg twice daily. The patient received a short course of prednisolone at 40 mg per day for five days before admission. The physical examination showed a central stridor during both inspiration and expiration. Chest radiograph showed a normal lung parenchyma and no hilar enlargement. Spirometry revealed fixed airway obstruction. CT scan of the thorax revealed a 2.4 × 2.7 cm lobulated mass abutting the right side of the lower trachea with nearly complete obstruction. Due to the large tumor causing significant central airway obstruction, the medical team opted to remove the central airway mass through rigid bronchoscopy. Argon plasma coagulation was used to facilitate mass shrinkage. Mechanical mass removal was performed using a rigid bronchoscope. At the end of the treatment, re-evaluation by bronchoscopy exhibited no remaining mass. Histologic examination conﬁrmed the diagnosis of a tracheal adenoid cystic carcinoma. No recurrence of the tumor was noted during 12 months of follow-up.

## Introduction

Primary tumors in the trachea are exceptionally uncommon, occurring in approximately 0.1 per 100,000 individuals annually. They constitute around 0.2% of all tumors in the respiratory tract and a mere 0.02% to 0.04% of all malignant tumors. In adults, the majority of tracheal tumors are cancerous, while only 10% to 30% of tracheal tumors in children are malignant [1]. Adenoid cystic carcinoma (ACC) is the second most common primary tracheal tumor in adults, following squamous cell carcinoma (SCC) [2]. ACC grows slowly, tends to recur, and obstructs the airway, causing worsening dyspnea. Treatment involves surgical removal of the affected tracheal portion and tumor with end-to-end anastomosis. Postoperative radiotherapy is recommended for incomplete resections [3]. However, there is limited data on ACC cases that have achieved success solely through bronchoscopic treatment. Here, we report successful treatment of tracheal ACC by rigid bronchoscopy to core out the tumor, combined with cryoextraction and argon plasma coagulation (APC), without recurrence of the tumor.

## Case presentation

**Figure 1:**
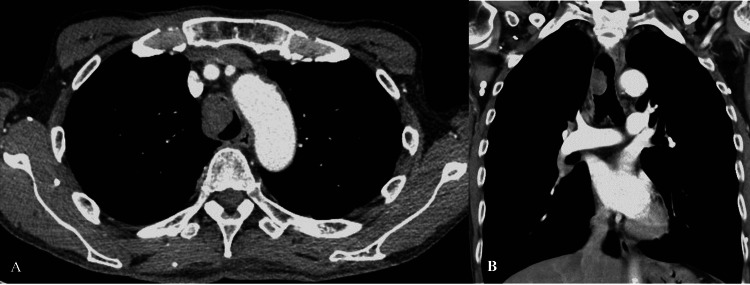
CT of the chest demonstrating a 2.4 × 2.7 cm lobulated mass abutting the right side of the lower trachea with nearly complete obstruction (A: axial view, B: coronal view).

**Figure 2:**
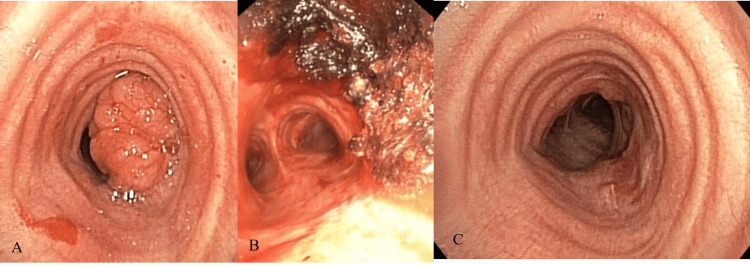
(A) Bronchoscopy showing a large lobulated mass at the right side of the tracheal wall with nearly complete tracheal lumen, (B) after immediate total tumor removal, (C) 12 months follow-up of bronchoscopy demonstrating healing of the excision site without evidence of tumor recurrence.

A 67-year-old male, ex-smoker of 25 pack-years, presented with mild dyspnea for two years, with increasing intensity and cough. He underwent outpatient evaluation and received treatment for recurrent episodes of bronchitis and acute exacerbations of chronic obstructive pulmonary disease. With the deterioration of the symptoms, he was admitted to the hospital, and a thoracic CT was performed, showing a 2.4 × 2.7 cm lobulated mass abutting the right side of the lower trachea with nearly complete obstruction (Figure 1). Bronchoscopy showed a large lobulated mass at the right side of the tracheal wall (Figure 2). It was located 2 cm above the carina. Due to the large tumor causing significant central airway obstruction, the medical team opted to remove the central airway mass through rigid bronchoscopy. APC was first used to facilitate mass devitalization. After the APC treatment, mechanical mass debulking using a rigid bronchoscope was successfully performed. Cryoextraction was performed to remove the fragmented tumors (Figure 2) and APC was used to coagulate the tumor base.

**Figure 3:**
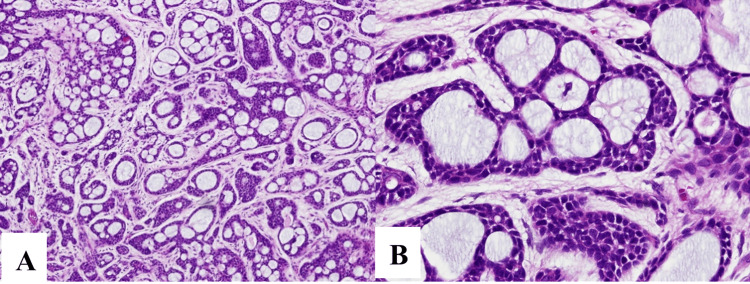
Pathologic macroscopic examination of the biopsy revealing a lobulated mass of 2.1 × 2.4 cm, microscopic examination of the biphasic salivary gland tumor composed of ductal and myoepithelial cells (hematoxylin and eosin (H&E) ×40) (A). The cribriform pattern is composed of predominantly myoepithelial cells with myxoid or hyalinized globules compatible with the diagnosis of a tracheal adenoid cystic carcinoma, cribriform subtype (H&E ×100) (B).

Macroscopic examination of the biopsy revealed a lobulated mass of 2.1 × 2.4 cm. Histological examination revealed a biphasic salivary gland tumor, comprising both ductal and myoepithelial cells. The cribriform pattern predominantly consisted of myoepithelial cells interspersed with myxoid and hyalinized globules, compatible with the diagnosis of a tracheal ACC, cribriform subtype (Figure 3).

Following bronchoscopic treatment, the patient was discharged the next day without any complications. Radiotherapy is often included in the treatment regimen for patients who are not candidates for surgery. However, in the presence of a purely intrinsic endobronchial tumor without regional lymph node involvement, the radiation oncologist suggested that radiation therapy was not necessary. In a follow-up bronchoscopy 12 months later, the excision site showed signs of healing, and there was no evidence of tumor recurrence (Figure 2). Neither the recurrence of dyspnea nor the tumor was noted during the one-year follow-up. Throughout the one-year follow-up period, the patient was evaluated every three months with chest X-rays and bronchoscopy to assess symptoms of dyspnea and hemoptysis and to evaluate the tumor base every three months. Chest CT performed after 12 months indicated no signs of tumor recurrence. Follow-up bronchoscopies at six and 12 months revealed minimal scarring and no evidence of recurrent disease. The patient reported improved symptoms with no dyspnea, hemoptysis, or recurrent pneumonia observed during the follow-up period.

## Discussion

Primary tracheal tumors are very rare, with an estimated annual incidence of 2.7 new cases per million, and typically exhibit malignancy in adults [1]. ACC is the second most common tracheal tumor arising from the minor salivary and serous glands, located in the tracheal submucosa. It is morphologically similar to primary salivary gland tumors [3]. ACC typically exhibits exophytic growth leading to the narrowing of the tracheal lumen. It is characterized by submucosal and perineural spread. At the time of diagnosis, up to 10% of patients had regional lymph node involvement or distant metastases [4]. Some ACC cases show slow progression, with a five-year survival rate of approximately 90%; however, long-term survival is less than 40% at 15 years. More aggressive cases are associated with advanced-stage neoplasms at diagnosis, resulting in higher recurrence rates and distant metastases. Recurrence, whether local or distant, can manifest after varying periods. ACC commonly develops in the fourth and fifth decades of life, with no gender predilection [5]. While the cause remains unknown, there is no association with smoking.

This patient unexpectedly received a diagnosis of tracheal ACC. He had dyspnea with intensity evolution and cough due to tracheal ACC. The primary symptoms of tracheal neoplasms arise from tumor obstruction. Dyspnea is a common manifestation, characterized by a gradual onset over several months, often accompanied by later development of stridor. Additional symptoms encompass wheezing, coughing, chest pain, and hemoptysis [6]. Diagnosing tracheal neoplasms in adults is often delayed by several months from the initial presentation. This delay may be attributed to the substantial functional reserve of the tracheal lumen, as tumors typically do not cause symptoms until they nearly occlude most of the luminal diameter. Non-specific symptoms such as cough, positional wheezing, and exertional dyspnea can lead to misdiagnoses such as asthma, chronic obstructive pulmonary disease, or bronchitis [7]. Exertional dyspnea typically becomes apparent only when the trachea has narrowed to less than 8 mm, and dyspnea at rest occurs when the lumen is less than 5 mm [8].

Bronchoscopy is the most valuable diagnostic tool for identifying tracheal cancer. It has a precise assessment of the nature and extent of the tumor. A biopsy may be conducted for pathological evaluation. The primary diagnostic and staging modalities for primary tracheal tumors encompass chest and neck CT scans in conjunction with bronchoscopy. CT scans play a crucial role in evaluating the depth of invasion, the potential involvement of adjacent structures, and detecting lymphogenic and distant metastases or primary synchronous lesions [9].

Histologically, cystic adenoid carcinoma is identified by a cribriform growth pattern, featuring cells with angulated nuclei and limited cytoplasm. Perineural invasion is a common characteristic. The tumors usually manifest as elongated, cylindrical formations lined with small cuboidal cells and containing deeply eosinophilic cores resembling basement membrane material. The previous classification of this tumor as “cylindroma” stems from these cylinder-like structures. The eosinophilic cores exhibit positive staining with periodic acid-Schiff, signifying the presence of mucinous material [10].

One study showed that ACC in the upper airway often exhibits local invasiveness into the wall but remains suitable for surgical resection. Both incomplete and, when feasible, complete resection have demonstrated satisfactory long-term outcomes for ACC patients [9]. In contrast to SCC, radiotherapy alone can achieve extended periods of remission [9]. Nevertheless, the optimal approach for maximizing survival in ACC patients involves surgical resection followed by radiotherapy [11].

The gold standard in treating tracheal ACC is a complete tracheal resection [11]. However, surgical resection is not a feasible option for malignant tumors involving the carina and/or distal trachea due to a difficult surgical approach. Rigid bronchoscopic intervention has been reported to successfully treat tracheal ACC for those unfit for surgery. Various methods including laser therapy, excision by bronchoscopic forceps, cryoextraction, and rigid coring were used to treat this tumor [10]. In our case, we preferred using rigid bronchoscopic intervention due to the patient’s refusal of surgery and the distal tracheal involvement. Tumor debulking was performed using the tip of the rigid bronchoscope and cryoextraction. APC was applied on the tumor bed before and after tumor removal by rigid bronchoscopy for coagulation and ablation of the residual tumor. The patient was discharged without complications the following day after the procedure with good oxygenation and no further dyspnea. Throughout the one-year follow-up period, a chest CT performed after 12 months indicated no signs of tumor recurrence. Follow-up bronchoscopies at six and 12 months revealed minimal scarring and no evidence of recurrent disease.

## Conclusions

Tracheal ACC is the second most common tracheal tumor and should be kept in mind for differential diagnosis among patients with progressive dyspnea and cough. Thoracic CT can identify tracheal tumors, and bronchoscopy is beneficial for both definite diagnosis and treatment. Rigid bronchoscopic intervention using combined rigid coring out, cryoextraction, and APC is a good treatment option among surgically unfit patients, without any significant acute and long-term complications.
